# Association between Sleeping Patterns and Mealtime with Gut Microbiome: A Pilot Study

**DOI:** 10.34172/aim.2022.46

**Published:** 2022-05-01

**Authors:** Zahra Mohammadi, Faraz Bishehsari, Sahar Masoudi, Azita Hekmatdoost, Delisha A. Stewart, Sareh Eghtesad, Maryam Sharafkhah, Hossein Poustchi, Shahin Merat

**Affiliations:** ^1^Liver and Pancreatobiliary Diseases Research Center, Digestive Diseases Research Institute, Tehran University of Medical Sciences, Tehran, Iran; ^2^Department of Internal Medicine, Division of Gastroenterology, Rush University Medical Center, Chicago, Illinois, USA; ^3^Department of Clinical Nutrition, School of Nutritional Sciences and Dietetics, Shahid Beheshti University of Medical Sciences, Tehran, Iran; ^4^Department of Nutrition, University of North Carolina at Chapel Hill, Nutrition Research Institute, Kannapolis, North Carolina, USA; ^5^Digestive Disease Research Center, Digestive Diseases Research Institute, Tehran University of Medical Sciences, Tehran, Iran

**Keywords:** Dietary behavior, Gut microbiome Mealtime, Sleeping pattern

## Abstract

**Background:**

Disruptions in sleep related to mealtime may contribute to gut microbial imbalances, and put individuals at higher risk for metabolic diseases. The aim of this pilot study was to investigate the relationships between late-night eating habits and sleep quality and duration, with gut microbiota (GM) profiles.

**Methods:**

In this cross-sectional study, 36 men referred to a clinic were enrolled. In addition to demographic information, each participant completed questionnaires regarding medical history, physical activity, late-night eating habits, sleep quality and sleep duration. The scores from these questionnaires were used to categorize study participants into the following groups: sleep quality (good or poor), late-night eating (yes or no) and sleep duration (<7 or ≥7 hours). Five grams of stool was also obtained from each participant for GM profiling analysis by sequencing.

**Results:**

The mean age of the study population was 42.1 ± 1.6 years. *Firmicutes* and *Actinobacteria* were the two dominant phyla present in all participant samples. Differences in the relative abundance of GM at each taxonomic rank between study groups were insignificant. Only *Erysipelotrichales* at the order level were found to be significantly different between individuals who had late-night eating habits and those who did not (*P* & *q* < 0.05). No other parameter demonstrated a significant difference in GM profiles of participants.

**Conclusion:**

In this pilot study, we found *Erysipelotrichales* to be more abundant in individuals with late-night eating habits. Studies with higher sample sizes are warranted to better delineate the possible effects of time of eating on microbial composition.

## Introduction

 Circadian rhythms (CR) represent the body’s 24-hour internal clock that regulates sleep-wake cycles in response to changes in light and darkness of the environment.^1^ Recently, several studies have shown that any disruption in CR, including feeding behavior and sleep patterns, can play a crucial role in human health as relates to the development of a wide spectrum of diseases, such as hypertension, diabetes, and cancers.^2-5^ The effect of CR disruption on the gut microbiome (GM) is still controversial and widely discussed by researchers around the world.^6^ Host CR does influence GM composition, which is conversely critical for the regulation of circadian pathways.^7,8^ The interaction between GM and CR can affect host physiological processes such as the immune response, endocrine regulation, and metabolism, subsequently affecting susceptibility to several diseases.^9-11^

 Recent studies have shown that GM and their metabolites change rhythmically throughout the day and night and are often influenced by the feeding-fasting cycle, feeding behavior, and sleep-wake cycle.^10,12,13^ Association between sleep patterns and GM composition has been studied extensively, where microbiome diversity has been associated with sleep quality as well as sleep time.^13,14^ Furthermore, it has been shown that changes in sleep duration can lead to changes in GM. Besides sleep, various human and animal studies have also evaluated the association between dietary behaviors and microbiome composition showing that feeding behaviors such as meal timing and frequency, meal skipping, and duration of overnight fast may affect the abundance and composition of GM.^12,15,16^ The aim of this study was to investigate the relationship between sleeping patterns, eating behaviors and GM profiles. We hypothesized that GM composition will be different in subjects with good versus poor sleep quality, shorter (< 7 hours) versus longer (≥ 7 hours) sleep duration and late-night versus not eating behaviors.

## Patients and Methods

###  Study Population

 Thirty-six men, referred to the Tehran Gastroenterology and Hepatology Clinic, were enrolled in the study. Given that animal and human studies have shown sex-related differences in gut microbiome, only men were included in this pilot, to limit confounding factors within the already small sample size.^17^ Males older than 18 years of age and willing to participate were included in the study, unless they met any of the following exclusion criteria: a history of autoimmune, metabolic, or gastrointestinal diseases, surgeries resulting in a change in gut anatomy, alcohol use at ≥40 g/wk, current use of corticosteroids or probiotics, recent use of vitamin E, fish oil supplements (within 6 months of recruitment) or antibiotics (within 6 weeks of recruitment) and finally, dieting within 1 month of recruitment. Upon entering the study, all participants signed a written informed consent.

###  Data and Sample Collection

 Using a 34-item questionnaire, demographic information including age, past and present medical history, surgical history, history of alcohol consumption, and smoking status were collected. Body mass index (BMI) and waist-to-hip ratio (WHR) were also measured and recorded. Table l summarizes this information for the study participants. To assess CR, a researcher-designed questionnaire was completed for workdays and weekends/holidays, separately. This novel questionnaire, developed and approved through discussions by a five-member expert panel in the field of CR, includes 11 items regarding the time of meals as well as wake and sleep times. Indices on sleep duration (<7 or ≥7 hours) and late-night eating habits defined as eating ≤2 hours before sleep (yes or no) were calculated based on this questionnaire (Supplementary file 1, Table S1).

 The Pittsburgh Sleep Quality Index (PSQI), previously validated in Farsi (Persian), was also measured for participants. The PSQI consists of 19 items to assess the quality of sleep in the month prior to its completion. Sleep quality is determined through the evaluation of various factors, including latency in falling sleep, duration of sleep, sleep disturbances, habitual sleep efficiency, use of sleeping medication, and daytime dysfunction.^18^

 To measure dietary intake, a 90-item qualitative food frequency questionnaire (FFQ) was completed. Individuals were asked about their usual consumption of common food items over the year prior to the interview. Nutrient information was obtained using the United States Department of Agriculture (USDA) food composition databases.^19^

 Clinical chemistries including serum fasting blood sugar (FBS), total cholesterol, triglycerides (TG), very-low-density lipoprotein (VLDL), high-density lipoprotein (HDL), insulin and hemoglobin A1c (HbA1c) levels were measured by standard kits. Approximately 5 grams of stool was collected from each participant and stored into a sterile 20-milliliter polypropylene fecal container and immediately frozen to -80°C.

###  Microbial Analyses 

 Fecal bacterial DNA was extracted using the FavorPrep TM Stool DNA Isolation Mini Kits (FAVORGEN, Taiwan) following the manufacturer’s protocol. DNA concentrations of samples were evaluated by Nanodrop (IMPLEN, Germany). After extraction, the V4 regions of microbial small subunit ribosomal RNA genes were amplified with primers CS1_515F and CS2_806R using Access Array Barcode Library for Illumina (Fluidigm, South San Francisco, CA; Item# 100-4876). The amplicons were produced in two steps.^20^ Fluidigm sequencing primers to target the CS1 and CS2 linker regions were used to initiate sequencing. Sequencing was performed at the Chicago Genome Research Core (GRC) within the Research Resources Center (RRC), University of Illinois, Chicago, USA. The standard QIIME pipeline, as a high-throughput sequencing technique, was modified to generate taxonomic summaries using sub-Operational Taxonomic Units (sub-OUTs).^21,22^ All sequences with an abundance of ≥10 counts were designated seed sequences. USEARCH was then used to find the nearest seed sequence for any non-seed sequence with a minimum identity threshold of 97%. For any non-seed sequence that matched a seed sequence, its counts were merged with the seed sequence counts.^23^ Taxonomic annotations for seed and unmatched non-seed sequences were assigned using the USEARCH and Silva v132 reference with a minimum similarity threshold of 90%.^23,24^ After quality filtering, beta and alpha diversity were calculated.^25,26^

###  Statistical Analysis

 Descriptive analyses were conducted using Stata version 12. Mean (SD) and median (range) were calculated for normal and skewed variables, respectively. Differential analyses of taxa as compared with experimental covariates i.e., sleep quality, late-night eating, and sleep duration were performed using R statistical software, edgeR package, on relative abundance.^27^ Prior to analysis, taxa with less than 0.1% of the total sequence abundance were removed. Normalized data were tested using the Wilcoxon test by experimental covariates. *P* values were corrected using the Benjamini-Hochberg false discovery rate (FDR) correction. A corrected *P* value (q value) less than 0.05 was considered statistically significant.

 For alpha diversity (the richness of different species in a sample), Simpson indices were calculated using default parameters in the vegan library of R software.^25^ The resulting Simpson indices were then modelled with the sample covariates using a generalized linear model assuming a Gaussian distribution and tested for significance by *t* test. Plots were generated through the ggplot2 library in R.^26^ To estimate beta diversity (different microbial communities in different environments), Bray-Curtis indices were calculated with default parameters in R using the vegan library.^25^ The resulting dissimilarity indices were modelled and tested for significance with the sample covariates using the ADONIS test. Plots were generated in R using the ggplot2 library.^26^

## Results

 Thirty-six participants were recruited for this pilot study. The mean age of study participants was 42.1 ± 1.6 years. The descriptive characteristics of participants are presented in Table 1. Results obtained from each questionnaire were used to categorize individuals into various groups and study variables were assessed among them. Based on the PSQI results, participants were divided into the following two groups: those with good sleep quality (n = 9) and those with poor sleep quality (n = 27). BMI was the only variable that was significantly different between the two sleep quality groups (*P *˂ 0.05). The sleep duration index extracted from the CR questionnaire was the basis of the second group categorizations including: individuals with less than 7 hours of sleep (n = 23), and those with ≥ 7 hours (n = 13). The third group classification, also based on the CR questionnaire, divided individuals into those with (n = 13) and without (n = 23) late-night eating behaviors. No other significant differences in baseline variables (Table 1) were observed between individuals for any of these two category groups.

**Table 1:** Descriptive Characteristics of the Participants

**Variables **		**Sleep Quality**		**Late-Night Eating**		**Sleep Duration**		**Total **
		**Good (n = 9)**	**Poor (n = 27)**	**Yes (n = 13)**	**No (n = 23)**	**<7 (n = 23)**	**≥7 (n = 13)**	**n = 36**
Age, (mean ± SD), years		37.7±2.4	43.6±2.0	40.08±3.0	43.2±2.0	40.6±2.0	44.7±2.7	42.1±1.6
BMI, (mean ± SD), (kg/m2)		32.5±3.8*	26.1±0.7*	27.8±2.3	27.7±1.3	28.1±1.7	27.0±1.1	27.7±1.2
WHR, (mean ± SD)		0.95±0.02	0.94±0.02	0.96±0.2	0.92±0.02	0.93±0.01	0.95±0.03	0.94±0.01
Systolic blood pressure, (mean ± SD)		114.5±4.8	116.8±3.1	122.3±5.7	112.6±2.3	117.0±3.7	114.6±3.0	116.1±2.6
Diastolic blood pressure, (mean ± SD)		77.8±4.0	79.9±2.4	82.3±4.6	76.5±1.7	79.6±2.5	77.0±3.3	78.6±2.0
Calorie intake, (mean ± SD)/in a week		25355.5±1256.4	24381.5±657.0	24558.0±808.3	24663.0±796.5	24533.0±784.5	24787.7±843.6	24625.0±579.4
PHA (%)	Low	11.1	22.2	23.1	17.4	13.0	30.8	19.4
	Medium	77.8	59.3	61.5	65.2	69.6	53.8	63.9
	High	11.1	18.5	15.4	17.4	17.4	15.4	16.7
Smoking (%)	Yes	44.4	29.6	23.1	39.1	34.8	30.8	33.3
	No	55.6	70.4	76.9	60.9	65.2	69.2	66.7
Insulin, median (min-max), micIU/mL		8.4 (5.7–23.7)	9.7 (3.5–15.0)	8.7 (4.4–23.7)	9.7 (3.5–23.0)	8.7 (3.5–23.7)	9.7 (4.4–14.3)	9.7 (3.5–23.7)
FBS, median (min-max), mg/dL		91.0 (86.0–224.0)	93. 0 (72. 0–198. 0)	92.0 (88.0–107.0)	93.0 (72.0–224.0)	93.0 (72.0–224.0)	92.0 (88.0–198.0)	92.5 (72.0–224. 0)
Cholesterol, median (min-max), mg/dL		168.0 (104.0–200.0)	166.0 (122.0–239.0)	159.0 (128.0–191.0)	168.0 (104.0–239.0)	168.0 (104.0–239.0)	166.0 (148.0–201.0)	167.0 (104.0–239.0)
TG, median (min-max), mg/dL		100.0 (76.0–294.0)	108.0 (45.0–386.0)	113.0 (59.0–294.0)	100.0 (45.0–386.0)	108.0 (56.0–386.0)	99.0 (45.0–294.0)	106.5 (45.0–386.0)
HDL, median (min-max), mg/dL		36.0 (29.0–44.0)	33.0 (26.0–59.0)	31.0 (27.0–41.0)	35.0 (26.0–59.0)	34.0 (26.0–59.0)	33.0 (28.0–49.0)	33.5 (26.0–59.0)
VLDL, median (min-max), IU/L		20.0 (15.2–58.8)	21.6 (9.0–77.2)	22.6 (11.8–58.8)	20.0 (9.0–77.2)	21.6 (11.2–77.2)	19.8 (9.0–58.8)	21.3 (9.0–77.2)
HbA1C, median (min-max), %		5.3 (4.7–9.7)	5.2 (4.5–8.8)	5.0 (4.5–5.6)	5.4 (4.7–9.7)	5.2 (4.5–9.7)	5.3 (4.8–8.8)	5.2 (4.5–9.7)

BMI, Body mass index; WHR, Waist to hip ratio; PHA, Physical Activity; FBS, Fasting blood sugar; TG, Triglycerides; HDL, High-density lipoprotein; VLDL, Very-low-density lipoprotein; HBA1c, Hemoglobin A1c. *P value less than 0.05.

###  Microbial Composition

**Figure 1:**
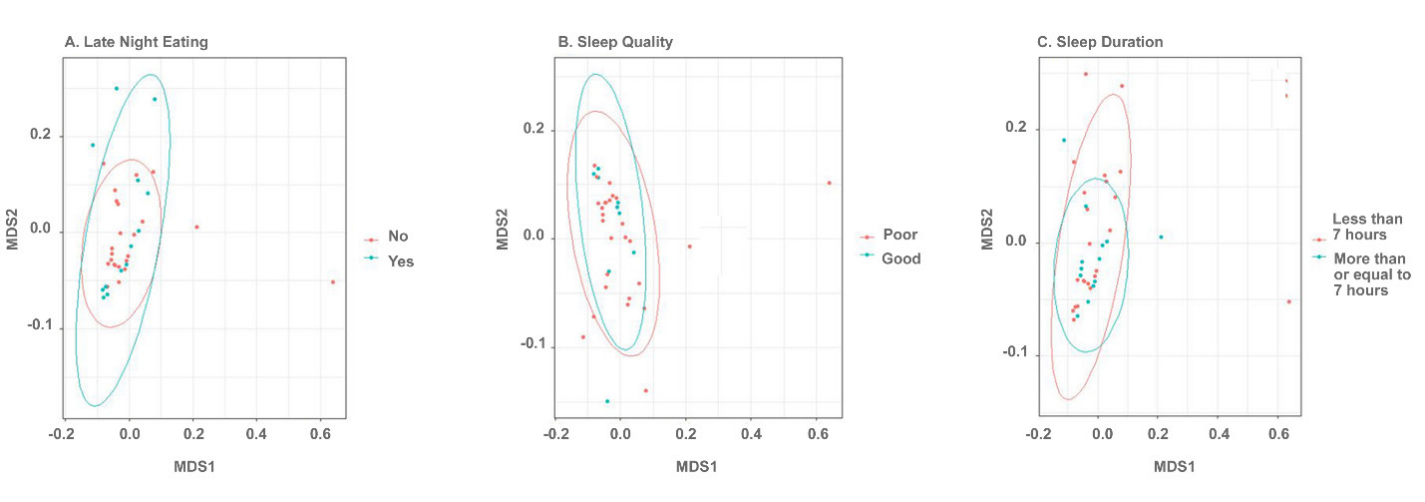
Non-metric Multi-dimensional Scaling (NMDS) plot of Bray-Curtis Dissimilarity Indices Computed Using the Taxonomic Summary Data at the Phyla Level. Points are colored based on sample group. **(A)** Late-night eating, **(B) **Sleep quality and **(C)** Sleep duration. Beta diversity was not significantly different at the phyla level.

**Figure 2:**
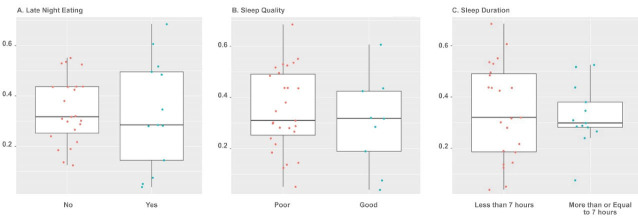
Dot and Box Plots of Alpha Diversity as Compared with Sample Groups. Points are colored based on sample group. **(A) **Late-night eating, **(B)** Sleep quality and **(C)** Sleep duration. Alpha diversity was not significantly different at the phyla level.

**Figure 3:**
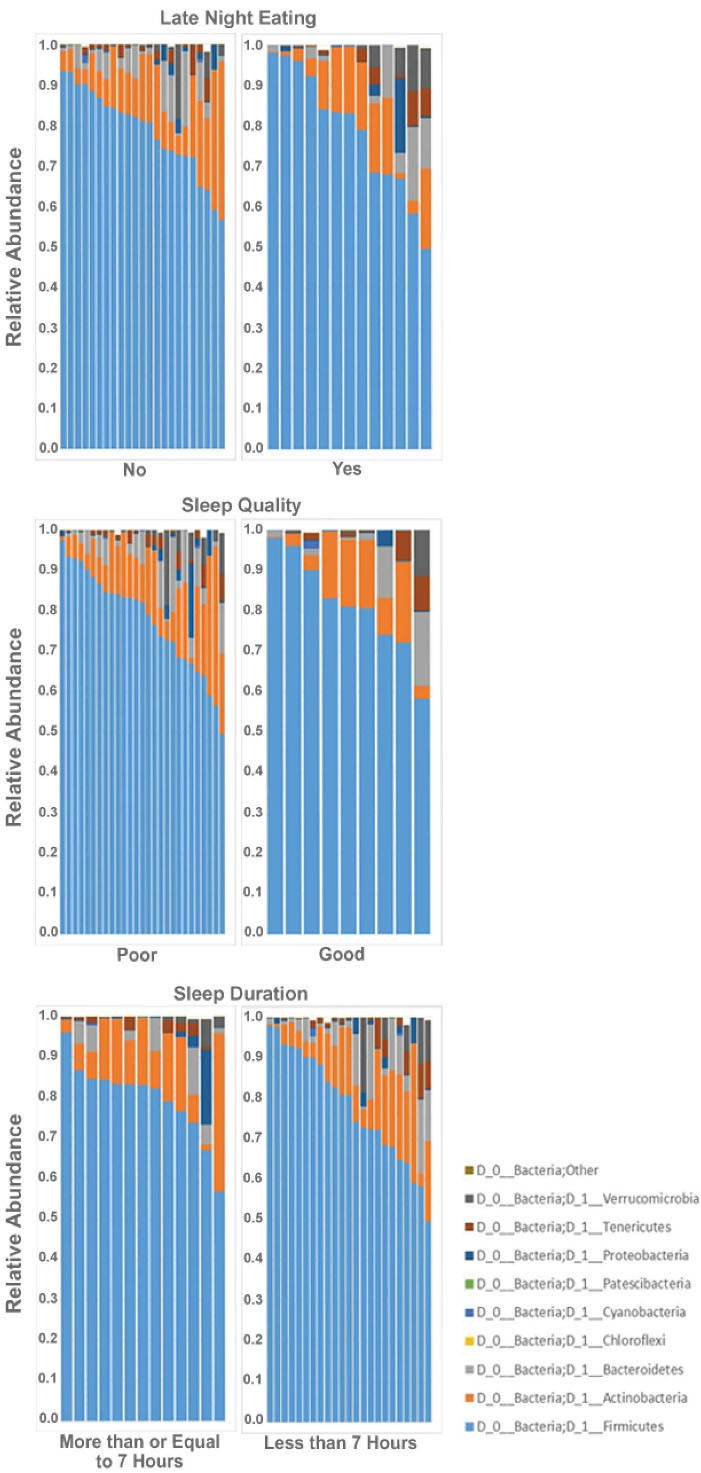
Phyla distribution (Relative Abundance) is shown for each participant sample based on study groups**. (A)** Late-night eating, **(B)** Sleep quality and **(C) **Sleep duration.

 Beta and alpha diversities at the phyla level were assessed with “ADONIS” and “Simpson” methods, respectively. As shown in Figures 1 and 2, there was no significant difference at the phyla levels between the study groups. The dominant phyla present in all participant samples were *Firmicutes* and *Actinobacteria*. The distribution of dominant Phyla in the participants based on study groups is shown in Figure 3. The relative abundance of GM at the phyla, class, order, family, genus, and species levels across study groups was insignificant; with the exception of *Erysipelotrichales* (order), which were significantly more abundant (mean ± SD, 0.05 ± 0.2 vs. 0.02 ± 0.03) in individuals with late-night eating behaviors compared to those without (*P* = 2.9306E-06, q = 7.91262E-05).

## Discussion

 Our study has demonstrated for the first time that *Erysipelotrichales* were more common in individuals who had late-night eating behaviors, compared to those who did not. Our results showed no significant differences in GM at the levels of alpha and beta diversity between the participants who sleep less than 7 hours compared to those who sleep more than 7 hours. We also found no differences in GM diversity between participants who reported having good sleep quality versus those who were scored as having poorer sleep quality. Previous studies have shown late-night eating to be associated with obesity,^28-30^ and bacteria of the family *Erysipelotrichaceae* were reported to be associated with metabolic disorders such as obesity.^31-32^ Zhang et al also reported a population shift for *Erysipelotrichaceae* in post-gastric-bypass obese individuals, indicating the GM alteration due to surgery and differences in food consumption and digestion perturbations.^33^ In addition, Kaczmarek et al demonstrated that the GM profiles for a number of gut bacteria exhibited an oscillatory behavior in response to the time of eating.^12^ For instance, it has been observed thatbacterial quantity in mice peaks at 11 pm (with a maximum in the *Bacteroidetes* population) and reaches a low at 7 am (with a maximum in the *Firmicutes* population), suggesting presence of a “bacterial clock” which is orchestrated in tandem with the host clock.^34^

 All groups in the present study were characterized by a similar distribution of age, past and present medical history, surgical history, history of alcohol consumption, smoking and waist to hip ratio, allowing for any differences observed in GM to be attributed to the comparison groups. Our main limitations were the small sample size and the assessment of only fecal GM and not the mucosal-related microbiota. Although mucosal specimens allow for better detection of bacteria, fecal specimens are non-invasive and easily obtainable, therefore suitable samples for evaluating GM profiles for this type of study.^35,36^ Studies on GM diversity in different populations improve our general knowledge about the effects of GM and their related metabolic pathways, on human health, as well as their association with a variety of diseases. The resultant knowledge can be generalized to inform for translation into treatments for patients via modulating the GM profile. Understanding the association between GM and CR holds the potential for microbiota-directed therapies such as use of probiotics and prebiotics to improve the effects of disrupted circadian homeostasis.

 In conclusion, in this study, we aimed to investigate the relationship between circadian phenotypes (sleeping and eating behaviors) with profiles of GM. We found that *Erysipelotrichales*, of *firmicutes*, previously linked to metabolic disorders and obesity, are more abundant in individuals who had late-night eating habits. This provides a targetable mechanism to ameliorate metabolically-associated diseases in high-risk individuals. Further studies with larger sample sizes are needed to confirm our results.

## Supplementary Materials

Supplementary file 1 contains Table S1.
