# Supplementary material for: Association between Sleeping Patterns and Mealtime with Gut Microbiome: A Pilot Study
Source: Arch Iran Med. 2022 May 1;25(5):279–84. doi: 10.34172/aim.2022.46 (PMC11904296; doi:10.34172/aim.2022.46)
Supplement: Supplementary file 1 — contains Table S1. [file aim-25-279-s001.pdf]

Supplementary file 1

Table S1. Circadian Rhythm Questionnaires.

Typical Work/School Day	Typical Free Day
What time do you usually wake up on these days?	
____ AM/PM	____ AM/PM
About what time do you usually eat the following meals or snacks each day?	
Breakfast ____ AM/PM	Breakfast ____ AM/PM
Snack 1 ____ AM/PM	Snack 1 ____ AM/PM
Lunch AM/PM	Lunch AM/PM
Snack 2 AM/PM	Snack 2 AM/PM
Dinner ____ AM/PM	Dinner ____ AM/PM
Snack 3 ____ AM/PM	Snack 3 ____ AM/PM
Which eating event (meal or snack) is usually the largest on each day?	
___Breakfast<br>___Snack<br>___Lunch<br>___Snack<br>___Dinner<br>___Snack	___Breakfast<br>___Snack<br>___Lunch<br>___Snack<br>___Dinner<br>___Snack
What time do you usually try to fall asleep on these days?	
____ AM/PM	____ AM/PM
Do you usually wake up in the night and eat on any of these days?	
Yes No	Yes No
If yes, what time?	If yes, what time?
____ AM/PM	____ AM/PM
What time do you have your bowel movements on these days?	
____ AM/PM	____ AM/PM
____ AM/PM	____ AM/PM
____ AM/PM	____ AM/PM
